# Glyco-oxidation and cardiovascular complications in type 2 diabetes: a clinical update

**DOI:** 10.1007/s00592-012-0412-3

**Published:** 2012-07-05

**Authors:** Francesco Piarulli, Giovanni Sartore, Annunziata Lapolla

**Affiliations:** Department of Medicine - DIMED, University of Padova, Via dei Colli 4, 35143 Padua, Italy

**Keywords:** Type 2 diabetes, Glyco-oxidation, Receptor for advanced glycation end products, Cardiovascular complications

## Abstract

Diabetes is associated with a greatly increased risk of cardiovascular disease (CVD), which cannot be explained only by known risk factors, such as smoking, hypertension, and atherogenic dyslipidemia, so other factors, such as advanced glycation end-products (AGEs) and oxidative stress, may be involved. In this frame, hyperglycemia and an increased oxidative stress (AGE formation, increased polyol and hexosamine pathway flux, and protein kinase C activation) lead to tissue damage, thus contributing to the onset of cardiovascular complications. Several studies have identified in various cell systems, such as monocytes/macrophages and endothelial cells, specific cellular receptors (RAGE) that bind AGE proteins. The binding of AGEs on RAGE induces the production of cytokines and intracellular oxidative stress, thus leading to vascular damage. Soluble RAGE levels have been identified as hypothetical markers of CVD, but, in this regard, there are sparse and conflicting data in the literature. The purpose of this review was to examine all the available information on this issue with a view to clarifying or at least highlighting the points that are still weak, especially from the point of clinical view.

## Introduction

Diabetes is associated with a two- to fourfold increase in the risk of coronary, cerebral, and peripheral artery disease. Seventy to eighty percent of people with diabetes die of atherosclerosis-related disease [1, 2]. Atherosclerosis develops much earlier in life and progresses at a faster rate, in diabetic patients than in individuals without diabetes. Despite the high prevalence of risk factors, no more than 25 % of the excess cardiovascular risk in diabetes can be attributed to known risk factors, for example, smoking, hypertension, and atherogenic dyslipidemia [3], so other factors, such as advanced glycation end-products (AGEs) [4–6], oxidative stress [7], and thrombophilic state [8], may be involved. In this sense, hyperglycemia is an important contributor to the onset of cardiovascular complications. Among the biochemical alterations characteristic of hyperglycemia, the factors involved in causing atherosclerotic disease include the formation of AGEs, an increased polyol pathway flux and hexosamine pathway flux, and protein kinase C activation [9–12]. All of these molecular mechanisms reflect a single hyperglycemia-induced process of superoxide overproduction by the mitochondrial electron transport chain. Hyperglycemia and an increased oxidative stress [13] thus lead to tissue damage via common pathways.

While the role of AGEs and their capacity to predict cardiovascular disease (CVD) in type 2 diabetic patients is now recognized, the published literature on other hypothetical markers, such as sRAGE and esRAGE, is sparse and conflicting. The reason why the picture is still not clear probably lies in the fact that soluble RAGE levels in humans depend on two mechanisms, that is, alternative splicing of the RAGE gene (esRAGE) and cleavage of the full-length protein (sRAGE).

The primary purpose of this review was to examine all the available information on this issue with a view to clarifying or at least highlighting the points that are still weak and need further investigation and clinical insight to arrive at a synthesis and interpretation of the controversial data in the literature.

### AGE formation

The non-enzymatic glycation of proteins, or Maillard reaction, is a process that links chronic hyperglycemia to a series of physiopathological changes considered important in the development of the chronic complications of diabetes [14]. The Maillard reaction is divided into three main stages: early, intermediate, and late.

In the “early stage,” glucose (or other reducing sugars, such as fructose, the pentoses, galactose, mannose, ascorbate, and xylulose) reacts with a free amino group of several molecules, including proteins, nucleic acids, and lipids, to form an unstable aldimine compound, the Schiff base. Through rearrangement, this base gives rise to a stable ketoamine, the Amadori product. Since this reaction does not require the participation of enzymes, the variables regulating it in vivo are the glucose and protein concentrations, the protein’s half-life and its reactivity in terms of free amino groups, and cell permeability to glucose. Under in vivo conditions, the Amadori product reaches an equilibrium after approximately 15–20 days, and, through irreversible links, it accumulates on both short-lived and long-lived proteins.

In the “intermediate stage,” involving oxidation and dehydration reactions, the Amadori product degrades into a variety of carbonyl compounds (glyoxal, methylglyoxal, deoxyglucosones) much more reactive than the sugars from which they derive; these compounds act as propagators, again reacting with the free amino groups of proteins. In particular, methylglyoxal is a highly reactive alpha-oxaldehyde formed both from reactions that depend on the glucose levels (non-enzymatic glycation, the polyol pathway) and from intermediate products of glycolysis, ketone body metabolism, and threonine catabolism. The marked reactivity and high plasma concentrations of methylglyoxal indicate that this is one of the most important compounds in vivo [15].

In the “late stage,” these propagators react again with free amino groups, and through oxidation, dehydration and cyclization reactions, they form yellow–brown, often fluorescent, insoluble, irreversible compounds (usually called AGEs), which accumulate on long-lived proteins and cause damage. Although the chemical nature of these compounds has yet to be clearly defined, recent investigations indicate that they include post-Amadori products deriving from oxidation and further structural rearrangements, so compounds that are neither cross-linked nor fluorescent have been assumed to belong to the AGE group. It should be emphasized here that oxidation processes are important in the formation of many AGEs [16]. There are two mechanisms behind these processes, both catalyzed by metals such as copper and iron: the first involves the auto-oxidation of free sugar in the presence of oxygen and free metals, leading to more reactive dicarbonyl compounds that react with proteins to form highly reactive ketoamines; the second mechanism involves protein-bound products of the Amadori pattern that are oxidized in the presence of oxygen and free metals, giving rise to highly reactive protein-enediols and protein-dicarbonyls, which can generate AGEs.

## Markers of glycoxidation

The change in color seen in glycated products in early studies prompted the use of spectroscopic methods (based on absorbance and fluorescence measurements) for their analysis, but these methods lacked specificity [14]. The more specific methods, based on HPLC, radioimmunoassay (RIA) and enzyme-linked immunosorbent assay (ELISA), subsequently used to study glycoxidation markers [17], have their limitations too, however, since they are unable to identify glycoxidation products structurally and they sometimes generate misleading results, as in the case of the first glycoxidation product identified—the FFI compound [18].

Mass spectrometry (a technique with a high sensitivity and specificity) has been applied more recently to studying glycoxidation in diabetic disease. Matrix-assisted laser desorption mass spectrometry (MALDI/MS) has identified a number of compounds after the in vitro incubation of protein with glucose [19, 20]; these compounds need to be tested under in vivo conditions, however, to confirm their clinical importance. MALDI/MS has also been applied successfully to the study of glycation and oxidation kinetics of such well-known proteins as albumin and globin, providing us with useful information about what happens in vivo [21]. A method based on LC/MS/MS was also used for the quantitative measurement of 16 biomarkers of protein glycation, oxidation, and nitration damage [22]. This investigation proved that hydroimidazolones are the most important glycoxidation biomarkers. In type 1 diabetic patients, the concentrations of protein residues modified by glycation show a twofold increase in hemoglobin A1 by comparison with healthy subjects. The plasma concentration of free adducts increases tenfold in the same patients, but no studies correlating these markers with chronic diabetic complications have been performed to date.

Among the AGEs, carboxymethyl-lysine (CML) has a well-characterized structure and is considered a glycoxidation product because a pro-oxidizing condition is needed for its formation; it is recognized by RAGE and capable of directly activating the AGE-RAGE-NF-kB axis [23]. It has been demonstrated that the CML formed by protein-bound glycated lysine oxidation is a major AGE in vascular lesions and that its level is associated with macrovascular complications in diabetic patients [24].

Pentosidine is a structurally characterized marker of cross-linking induced by glycoxidation; it is associated with early atherosclerosis in type 2 diabetic patients (increased carotid intima-media wall thickness [25] and arterial stiffening [26]), and also with advanced atherosclerosis, in term of peripheral artery disease (PAD) [27].

## The role of AGEs in diabetic macroangiopathy

AGEs are formed by the glycation or glycoxidation of proteins, lipids, and nucleic acid and have been linked to the accelerated atherosclerosis seen in diabetic patients.

There are essentially three main mechanisms behind the tissue damage caused by AGEs, that is, intracellular glycation, cross-link formation, and interaction with specific cellular receptors [28].

### Intracellular glycation

The first mechanism of AGE-induced damage is a consequence of hyperglycemia-induced intracellular accumulation (demonstrated in macrophages and in endothelial and smooth muscle cells) and the resulting alteration of cytoplasmic and nuclear structures, including proteins involved in regulating gene transcription [29]. The accumulating AGEs spread out of the cell, inducing non-receptor-dependent changes in the extracellular matrix molecules [30] and modifying circulating proteins, which then bind to AGE cell receptors; this binding prompts the production of inflammatory cytokines, which in turn cause vascular injury [31].

### Cross-link formation

This involves mechanisms classifiable as non-receptor dependent, of which one of the most important is the formation of stable abnormal cross-links on collagen, as demonstrated by in vitro incubation with glucose and also in vivo in the collagen of diabetic patients. The numerous chemical and physical changes to collagen caused by glycoxidation can explain some of the structural tissue modifications typical of the chronic complications of diabetes, such as vascular and arterial stiffening, and basement membrane thickening. The non-enzymatic glycation of collagen can also inhibit the release of endothelium-derived nitric oxide, with consequent vasoconstriction, reduced plasma flow, and tissue ischemia. Lastly, AGE compounds in collagen may trap multiple macromolecules such as lipoproteins, immunoglobulin, fibrin, and albumin. Immunoglobulins bound to collagen retain their ability to form antigen–antibody complexes, which may become deposited on the vessels. In addition, trapped immunoglobulin could trigger complement activation, resulting in tissue damage, and glycated LDL (AGE-LDL) also binds covalently to glycated collagen, thus contributing to vessel occlusion. Other mechanisms involved in the formation of atherosclerotic plaques may cause vessel occlusion too. For a start, AGEs (and particularly CML-LDL) [5, 32] are not identified by their receptor, they are preferentially recognized by scavenger receptors on monocytes/macrophages, and this enhances their uptake with a consequent stimulation of ester-cholesterol synthesis and “foam cell” formation. Second, glycated LDLs are capable of stimulating thromboxane β_2_ release and inducing platelet aggregation. Third, glycated lipoprotein may generate free radicals, prompting more vessel oxidative damage. It has been suggested that LDL glycation may increase susceptibility to oxidation [33] and thus contribute, together with small/oxLDL, to accelerating atherosclerotic processes. Finally, because of their altered structure, AGE-LDLs are immunogens and can therefore stimulate the production of antibodies, as well as oxLDL; the resulting immune complexes may deposit on vessel walls and stimulate “foam cell” formation (in the case of AGE-LDLs) or IgG oxLDL antibodies within atherosclerotic lesions, which are markers of an advanced stage of the atherosclerotic process [34].

### Interaction with specific cellular receptors

**Fig. 1:**
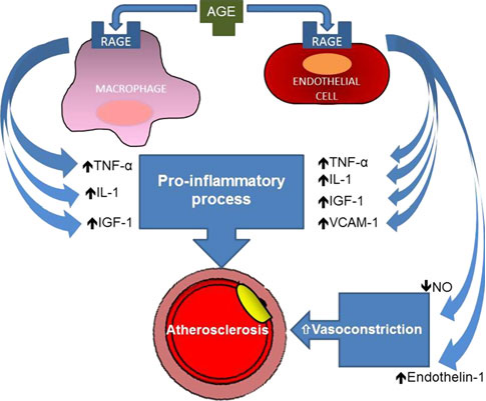
Cellular binding of advanced glycation end-products induces atherosclerosis. The mechanisms by which advanced glycation end-product (AGE) binding to specific receptors (RAGE) on macrophages and endothelial cells may cause atherosclerotic changes in diabetic blood vessels. On both cells, AGE-RAGE binding stimulates production of tumor necrosis factor (TNF)-α, interleukin-1 (IL-1), and insulin-like growth factor-1 (IGF-1) at levels that induce a pro-inflammatory process. On endothelial cells, AGE-RAGE binding induces increased expression of leukocyte-binding vascular adhesion molecule-1 (VCAM-1), increased intracellular oxidative stress and consequently reduced NO, and increased production of endothelin-1

Several studies have identified specific cellular receptors (RAGEs) that bind AGE proteins in a saturable manner. The RAGE was identified as a new member of the Ig superfamily of cell surface molecules, codified from a gene on chromosome 6. This receptor was subsequently identified in various cell systems, such as, monocytes/macrophages, T lymphocytes, fibroblasts, smooth muscle cells, endothelial cells, neurons, red cells, and mesangial cells. The binding of AGEs on RAGEs of T lymphocytes stimulates the production of gamma-interferon, with consequent tissue damage; the binding of AGEs to monocytes/macrophages (Fig. 1) induces the production of cytokines (interleukin 1B, TNF-alpha, IGF-1, PDGF) and growth factors, with a consequent increase in the synthesis of type IV collagen, a greater proliferation of vessel smooth muscle cells, and a stimulation of macrophage chemotaxis. Through a mechanism of oxidative stress, AGE-RAGE binding on endothelial cells induces the transcription factor NF-kB, which in turn increases the expression of the vascular cellular adhesion molecule (VCAM-1) (Fig. 1). The resulting VCAM-1 overexpression increases the adhesivity of monocytes to endothelial cells, and vascular permeability, speeding up the trans-endothelial passage of AGE-modified proteins (and AGE-LDL in particular). Other changes seen as a consequence of this AGE-RAGE interaction are an increased pro-coagulant response to TNF-alpha, a reduced thrombomodulin expression, and higher endothelin-1 levels. In diabetes, the binding of AGE to RAGE reduces tissue protein degradation and increases growth factor production, with a consequent increase in the synthesis of extracellular matrix and impairment of tissue remodeling mechanisms. In addition to oxidative stress, the absence of an appropriate compensatory response from the endogenous antioxidant network has also been implicated in systemic endothelial dysfunction [35, 36], of which microalbuminuria is considered a marker [37]. In this context, we found that microalbuminuria in type 2 diabetic patients might be promoted by an insufficient counter-regulation of the antioxidant system in the event of increased glycoxidation/glycation [38].

In short, AGEs contribute to endothelial damage via several mechanisms, including intracellular protein modifications, the formation of cross-links in the extracellular matrix, and interaction with the receptor for AGEs, that is, RAGE [39]. This receptor is a multiligand member of the Ig superfamily of cell surface molecules [40] that engages AGEs and leads to cellular signaling, including nuclear factor kB (NF-kB) activation, increased cytokine and adhesion molecule expression, the induction of oxidative stress [41], and an increase in cytosolic reactive oxygen species [42]. RAGEs may therefore have a part to play in the development of vascular disease in diabetic patients [43]. It is worth noting at this point that, in addition to the RAGEs, a number of RAGE ligands have been identified in diabetic patients, including members of the S100 calgranulin family and high-mobility group box 1 (HMGB1). These ligands’ interaction with RAGE can trigger a subsequent interaction with an innate immune system signaling molecule, the toll-like receptor 4 (TLR-4) [44].

The soluble RAGE isoform (sRAGE) expresses the cellular concentration of RAGE and reflects the total pool of soluble RAGE in plasma, which includes several variants. The exact role of sRAGE in plasma is unknown, but it may differ between different variants. Endogenous soluble RAGE (esRAGE) is a circulating truncated variant of the RAGE isoform that can neutralize AGE-mediated damage by competing with cell surface RAGE for ligand binding. EsRAGE is generated by the alternative splicing of the pre-mRNA RAGE transcript, and it is characterized by a specific C-terminal 16-amino-acid sequence [45]. EsRAGE accounts for most of the total soluble RAGE isoform (sRAGE) [46] detectable in the plasma using antibodies against full-length sRAGE (the three extracellular immunoglobulin domains) or antibodies specific for esRAGE. Antibodies against sRAGE recognize both sRAGE and esRAGE, but antibodies selectively reacting to esRAGE will not recognize other sRAGE-like molecules [47]. Plasma levels of esRAGE reflect receptor expression levels more directly than those of sRAGE [48].

## Metabolic memory

The results of the EDIC study [49, 50] on type 1 diabetic patients and those coming from the follow-up after the UKPDS study [51] on type 2 diabetic patients showed that patients who achieve a good metabolic control with intensive treatment when their diabetes is first diagnosed continue to benefit from a lower risk of chronic diabetic complications even if their glycemic control becomes worse. This is due to a phenomenon called “metabolic memory” or the “legacy effect” and can be explained by persistent epigenetic changes prompted by the increase in mitochondrial superoxide levels induced by hyperglycemia. Briefly put, hyperglycemia raises the mitochondrial production of ROS, and this activates the NF-kB subunit p65 in the proximal promoter with a consequent increase in the expression of p65-dependent proinflammatory genes. Activation of the proinflammatory genes of human aorta endothelial cells can induce macroangiopathy [44]. In parallel, the increased formation of stable AGE cross-links on collagen vessels can also contribute to the onset of micro- and macroangiopathy: in this frame, it has been suggested that hyperglycemia-induced functional and structural alterations in the microcirculation interact in the vascular continuum with larger arteries, that is, microvascular changes within the vessel wall can promote atherosclerosis in the larger arteries. Microvascular changes occurring in the first stage of diabetic disease could be reversible if the patient’s hyperglycemia is corrected promptly; if it is not, the resulting macrovascular changes are irreversible [52].

## AGEs and the AGE-RAGE axis: clinical implications in type 2 diabetes

AGEs play an important part in the development of cardiovascular complications in patients with type 2 diabetes. From the first observations of high AGE levels in atherosclerotic coronary plaque in selected type 2 diabetic patients [53] to Kiuki et al.’s demonstration that serum AGE concentrations increase consistently with the severity of coronary atherosclerosis in type 2 diabetic patients with obstructive coronary artery disease [54], the hypothesis that AGE concentrations may be a risk marker in type 2 diabetic patients with coronary atherosclerosis has taken shape and has been put to the test. A few years ago, the role of CML (one of the most often measured AGEs) was investigated in patients with ischemic heart disease (IHD) with and without type 2 diabetes [55]: patients with IHD and type 2 diabetes had significantly higher CML levels than diabetic patients without IHD. The authors concluded that the higher CML levels reflected the enhanced oxidative stress due to higher blood glucose levels in diabetic IHD patients. Data obtained in senile diabetic patients with and without cardiovascular complications appeared soon afterward [56] in a study in which serum AGEs were found significantly higher in senile diabetic patients with cardiovascular complications. The two studies suggested using serum CML or AGEs, respectively, as a useful independent biomarker of cardiovascular complications due to type 2 diabetes mellitus. There were no prospective data available at the time to confirm this recommended ion, however. A study confirming CML as a valid marker appeared 3 years ago, though it did not concern diabetic patients [57]. After a 6-year follow-up, Semba et al. found that plasma CML was an independent predictor of CVD-related and all-cause mortality in a population of elderly adults (without diabetes mellitus), without providing data on specific adverse cardiovascular outcomes, such as myocardial infarction or stroke. Table 1 lists the latest studies that move in this same direction, further supporting the association between glycoxidation and cardiovascular events or complications in type 2 diabetes. The results of these studies show a strong correlation between glycoxidation markers and the onset of complications, in terms of cardiovascular events or vascular damage in type 2 diabetes.

**Table 1:** The latest studies that support the association between glyco-oxidation and cardiovascular events or complications in type 2 diabetic patients

Author (year)	*n*	Type of study	AGEs	esRAGE	sRAGE	Complication
Kilhovd (2007)	386	Longitudinal	↑	–	–	Mortality (total, CVD, IHD) in women
Lapolla (2007)	33	Cross-sectional	↑	–	–	PAD
Yan (2008)	151	Cross-sectional	–	↓*	↑	CAD
Lu (2009)	357	Cross-sectional	–	↓	–	CAD
Peng (2009)	42	Longitudinal	–	↘	–	CAD progression
Basta (2011)	58	Cross-sectional	–	–	↑	IHD
Park (2011)	27	Cross-sectional	–	↔	↑	AMI
Colhoun (2011)	167	Longitudinal	–	↑	↑	IHD
Chen (2011)	58	Cross-sectional	↑	↓	–	PAD
Yang (2012)	41	Cross-sectional	–	↔↓	↔↓	Carotid plaque inflammation

*AMI* acute myocardial infarction, *CAD* coronary artery disease, *CVD* cardiovascular disease*, IHD* ischemic heart disease, *PAD* peripheral artery disease, *esRAGE* endogenous soluble receptor for AGE, *sRAGE* soluble receptor for AGE

* only esRAGE independently associated with CAD

↘ from baseline in diabetic patients with plaque progression

↔ similar between patients with and without AMI

↔↓ tenderness to be lower (P<0.07) vs healthy controls

Measuring serum levels of AGEs might pinpoint individuals at higher risk of cardiovascular complications. Increased serum levels of AGEs have been found to predict both cardiovascular and coronary mortality in women with type 2 diabetes, even during a follow-up spanning 18 years [58]. Very recently, the same results were seen by Nin et al. [59] in type 1 diabetic patients after a follow-up lasting 12 years. In type 2 diabetes, glycoxidation might contribute to the development of atherosclerosis not only in coronary arteries but also in the below-the-knee peripheral artery tree, as suggested by Lapolla et al. [27]. In particular, pentosidine levels were found much higher in type 2 diabetic patients with PAD than in cases without this condition, and pentosidine may be a predictor of PAD in type 2 diabetes. Certainly, the current data warrant an appropriately designed longitudinal study to confirm this hypothesis.

It is worth noting here that serum AGE levels do not necessarily correlate with fasting plasma glucose or recent glycated HbA1c levels, as Kilhovd et al. and Lapolla et al. have both observed; this is probably because the rate of AGE turnover is unrelated to glucose levels, whereas long-term poor glycemic control correlates with AGE production [54]. Even a long period of good metabolic control is unable to normalize the levels of glycoxidation products, such as pentosidine, showing that hyperglycemia causes a persistent oxidative stress that is able—per se and regardless of glucose concentrations—to induce and potentiate AGE formation in diabetic patients [60].

In hemodialysis patients too [61], plasma levels of AGEs, such as CML and pentosidine, have been associated with the levels of cardiac troponin T, a biomarker of myocardial damage used in the diagnosis of acute myocardial infarction and acute coronary syndrome. So, AGEs may be involved in the onset of myocardial damage in IHD patients too, including those with type 2 diabetes; AGEs accumulate in renal failure as well, due to their decreased excretion and increased generation caused by oxidative and carbonyl stress in uremia.

Four years ago, Basta [41] focused on the RAGE-ligand axis as an important player in modulating several steps of atherogenesis. AGEs induced oxidative stress through interaction with RAGEs. This has drawn a good deal of attention to the interaction between glycoxidation parameters and serum RAGE, which prompts an increased inflammatory reaction, supporting the pathogenic role of RAGE in the development and progression of atherosclerosis. Yan et al. [62] suggested that this interaction, together with the inflammatory cascade that follows, leads to coronary heart disease (CHD) in which the protective esRAGE levels are attenuated, as in type 2 diabetic patients. This picture was confirmed by Lu et al. [63] in a cross-sectional study and by Peng et al. [64] over a one-year period, who demonstrated an association between decreased esRAGE levels and coronary artery disease (CAD) severity or progression in patients with type 2 diabetes, respectively. Lower esRAGE levels, combined with an intensified inflammatory process, were associated with an accelerated atherosclerotic process. Here again, a thorough characterization of the protective function of esRAGE against plaque progression in T2DM patients is needed, based on serial observations over a long-term follow-up. More evidence of the association between esRAGE and diabetic cardiovascular complications in patients with T2DM has now become available. In particular, we ourselves [65] found significantly lower plasma esRAGE levels in diabetic patients with plaque than in those without plaque, along with higher levels of AGEs. We also made the original finding that esRAGE levels only correlated directly with glycoxidation parameters in patients with no plaque, suggesting that there are two different phenotypes of type 2 diabetic patients with a different susceptibility to glycoxidation. In patients without macrovascular complications, esRAGEs seem to have a vessel-protecting role and the capacity to neutralize glycoxidation.

An interesting recent study [66] found higher circulating sRAGE levels in patients with acute coronary syndrome and positive troponin I than in cases with normal troponin I. The study sample also included some type 2 diabetic patients, but their number was not stated. This was an observational study so no final conclusions can be drawn from it, but the authors hypothesized that acute myocardial injury, by inducing inflammatory reactions, could be a prominent stimulus for the release of sRAGE. Similar results in a prospective study on type 1 diabetic patients [67] reinforce the idea of a strong association between higher plasma sRAGE levels (as a reflection of RAGE expression) and fatal or non-fatal CVD-related events. In another recent study [68], the possible role of sRAGE and esRAGE in type 2 diabetic patients was extended to a subgroup analysis on patients with acute myocardial infarction (AMI). Plasma levels of sRAGE were higher in response to circulating AGEs in the type 2 diabetic patients with AMI than in the diabetic controls, and on multiple logistic regression analysis, they were independently associated with AMI. The plasma levels of esRAGE were similar in the two groups, however. The studies by Basta [66] and Park [68] help to clarify the role of sRAGE levels in type 2 diabetic patients, in whom they are positively and independently associated with the presence of CAD. In type 2 diabetes, sRAGEs lose their protective effect and their proinflammatory property prevails because of the specific state induced by the disease.

Two further studies have recently showed more light on the role of sRAGEs and esRAGEs in cardiovascular complications. A prospective study conducted by Colhoun et al. [69] showed that higher sRAGE and esRAGE levels are associated with future CHD, but not stroke, in type 2 diabetes. Another interesting finding that was not emphasized enough was that sRAGEs and esRAGEs seem to have no significant association with classical CVD risk factors such as smoking, lipids, blood pressure, or duration of diabetes, and no more than 6 and 10 % of the variance in sRAGE and esRAGE levels, respectively, was explained by BMI, ethnicity, HbA1c, or eGFR, which goes to show their independent role in predicting CHD events. In a cross-sectional study [70] examining a situation of frank cardiovascular damage, lower serum esRAGE levels and higher serum AGE levels were found associated with dyslipidemia and atherosclerosis in patients with type 2 diabetes. The authors attributed the decrease in serum esRAGE to three possible mechanisms: (1) chronic hyperglycemia directly inhibiting the synthesis and secretion of esRAGEs, (2) the accumulated AGEs binding to esRAGEs and leading to their greater clearance, and (3) inflammation interfering with esRAGE synthesis.

Park et al. [68] added a relevant observation that points in the direction of a close relationship between sRAGE and inflammation: they suggested that interactions between AGEs and their receptor RAGEs may be anti- or pro-inflammatory depending on the inflammatory milieu. In AMI, this mechanism ultimately results in macrophage infiltration at the site of atherosclerotic plaque and a worse inflammatory state, which destabilizes the fibrous cap tissue, enhancing the risk of rupture; this would be consistent with the finding of a RAGE overexpression associated with enhanced inflammatory reactions in the vulnerable region of the plaque in carotid endoarterectomy specimens [71]. A more severe carotid plaque inflammation in type 2 diabetic patients than in healthy controls has already been observed [72] using a specific technique to detect inflammatory carotid atherosclerotic plaque. Circulating sRAGE and esRAGE concentrations tended to be lower in the diabetic group, albeit without reaching statistical significance. A significant negative independent association between vascular inflammation and sRAGE levels was observed, but not between sRAGE and intima-media thickness (IMT), in all the subjects evaluated (both diabetic patients and healthy controls), making it difficult to establish the real association between this picture and diabetes. Considering that IMT provided no information on plaque composition or inflammatory state, the lack of association with sRAGE is not surprising.

**Fig. 2:**
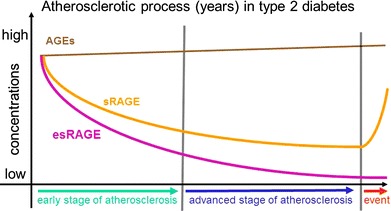
Behaviour of serum level of AGEs, sRAGE, and esRAGE in atherosclerotic process occurring in type 2 diabetes: a working hypothesis. AGE, soluble RAGE in a dynamic role, switching from higher levels in the early stages of inflammation and atherosclerosis to a gradual mild decline in the advanced stage of atherosclerosis, before increasing again in the acute phase of tissue damage(clinical events) and consequent inflammation

A recent study [73] providing new information on the correlation between glycoxidation factors like AGEs and pentosidine and diabetic cardiomyopathy supports the hypothesis that AGEs can induce subclinical diastolic dysfunction in type 2 diabetic patients. More importantly, it demonstrates that a decline in antioxidant defenses correlates closely with a worsening diastolic dysfunction. In this context, the interaction between glycoxidation and antioxidant status seems to play an important part in T2DM-related endothelial dysfunction, confirming previous results [38] (Fig. 2)

.

## Conclusion

Taken together, all the above-mentioned studies have shown that glycoxidation has a significant role as a mediator of T2DM-related cardiovascular complications, but it seems from the available evidence and from this review, that this role is only starting to be delineated. Both sRAGEs and esRAGEs have been suggested as biomarkers of CVD, but the studies conducted to date have formulated different hypotheses, and there is a paucity of prospective data. In particular, the crucial contribution of sRAGE to accelerated atherosclerosis through the activation of cellular inflammatory and proliferative processes needs to be clarified in further longitudinal studies with a view to confirming their capacity to predict the onset of cardiovascular complications, particularly in the carotid arteries. Although we are still far from a thorough understanding of serum level sRAGE behavior, we have to start thinking of RAGE in a dynamic role, switching from higher levels in the early stages of inflammation and atherosclerosis to a gradual mild decline in the intermediate stage, before increasing again in the acute phase of tissue damage and consequent inflammation. This dynamism may help to explain the different results obtained depending on the inflammatory conditions and also on the area or intensity of the damage. It is important here to bear in mind the previously mentioned hypothesis that the type 2 patient phenotype could be particularly important, since the expression of alternative RAGE splicing could be genetically determined, featuring a different susceptibility to glycoxidation.

Finding that type 2 diabetic patients have markedly decreased esRAGE levels and increased levels of its ligand (AGE), resulting in a significantly higher atherosclerotic burden, highlights the importance of esRAGE in the pro-atherogenic mechanisms at work in type 2 diabetes. EsRAGE might be an endogenous factor that protects against atherosclerosis and endothelial dysfunction mediated by oxidative stress, and part of the cell’s anti-oxidative defenses against vascular damage. The idea that their measurement should be recommended in type 2 diabetic patients needs to be tested in prospective studies on large samples in order to fully elucidate the relationship between esRAGEs and cardiovascular complications in type 2 diabetic patients.
